# Understanding How Parents Make Meaning of Their Child’s Behaviors During Screening for Autism Spectrum Disorders: A Longitudinal Qualitative Investigation

**DOI:** 10.1007/s10803-020-04502-7

**Published:** 2020-04-23

**Authors:** Thomas I. Mackie, Ana J. Schaefer, Leah Ramella, Alice S. Carter, Abbey Eisenhower, Manuel E. Jimenez, Angel Fettig, R. Christopher Sheldrick

**Affiliations:** 1 Department of Health Behavior, Society and Policy, Rutgers School of Public Health, 683 Hoes Lane West, Piscataway, NJ USA; 2 Institute for Health, Health Care Policy and Aging Research, Rutgers University, 112 Paterson Ave, New Brunswick, NJ 08901 USA; 3 Department of Health Law, Policy and Management, School of Public Health, Boston University, One Silber Way, Boston, MA 02215 USA; 4 Department of Psychology, University of Massachusetts Boston, 100 Morrissey Boulevard, Boston, MA USA; 5 Department of Pediatrics, Rutgers Robert Wood Johnson Medical School, 89 French Street, New Brunswick, NJ 08901 USA; 6 College of Education - Special Education, University of Washington, 2012 Skagit Lane, Box 353600, Seattle, WA 98195 USA

**Keywords:** Autism spectrum disorders, Screening, Qualitative, Parental perception

## Abstract

A family’s journey in understanding their child’s behaviors in relation to Autism Spectrum Disorders (ASD) frequently begins with screening. This study aimed to characterize the interpretive processes that unfold for parents. We employed longitudinal interviews with 19 families engaged in a community-based multi-stage screening protocol. Parents participated in 1–6 interviews dependent upon children’s length of engagement in the screening protocol; data were analyzed through modified grounded theory. Parents who moved towards understanding their child’s behaviors as ASD expressed (1) sensitization to ASD symptoms, (2) differentiation from other developmental conditions, and (3) use of the ASD diagnosis to explain the etiology of concerning behaviors. Identifying interpretive processes involved during ASD screening provides new opportunities for shared decision-making.

The Institute of Medicine/National Academy of Medicine (IOM/NAM) has emphasized the imperative of focusing on patients’ perspectives to reduce diagnostic errors, which are characterized not only by a failure to “establish an accurate and timely explanation of the patient’s health problems,” but also failure to “communicate that explanation to the patient” (National Academies of Sciences 2015, p. 35). Consistent with this definition, prior research on children with developmental disabilities suggests that how clinicians and parents arrive at a child’s diagnosis and the meaning the family makes of the diagnosis are influential to later service engagement, family coping and adaptation, and child outcomes (Moses 2010; Sher-Censor et al. 2019). During the process of screening and assessment for Autism Spectrum Disorder (ASD) (i.e., “screening period”), parents report experiencing confusion (Ryan and Salisbury 2012), stress, and anxiety (Midence and O’Neill 1999; Abbott et al. 2012). However, the extant literature draws relatively little attention to how screening tools and their administration influence parental perceptions of their child’s behaviors during this often uncertain and emotional time. The aims of the current study are to examine how parents “make meaning” of children’s behaviors as they engage in a multi-stage screening process for early identification of ASD. The present study specifically investigates how both the screening tools for ASD and their administration influence parents’ perceptions of their child’s behaviors moving them either towards or away from an interpretive frame consistent with symptomatic, diagnostic, or prognostic characteristics of ASD (hereafter, the “ASD interpretive frame”).

Recommendations for reducing diagnostic errors in medicine provided by the IOM/NAM emphasize the importance of shared decision-making between clinicians and patients when assessing whether to advance families towards diagnosis (National Academies of Sciences 2015). Elwyn et al. (2010) define shared decision-making as: “an approach where clinicians and patients share the best available evidence when faced with the task of making decisions, and where patients are supported to consider options to achieve informed preferences” (p. 1361). Prior research emphasizes the requirement for shared decision-making to rely on good clinical communication skills, especially in cases where patients and their families demonstrate low health literacy (Braddock et al. 1997; Elwyn et al. 2012). Screening tools and their administration may support communication with parents, including those with low health literacy, by providing an opportunity to carefully review the symptomatic presentation, diagnostic criteria, and prognosis associated with ASD. However, limited research to date examines whether and how screening tools and their administration incrementally alter the interpretive frame through which families’ perceive their child’s behaviors.

Literature on the experiences of parents largely focuses on a “loss” or “stress-reaction” paradigm, both of which tend towards investigation of how the ASD diagnoses impacts and presents new pressures to the family that require adaptation (Avdi et al. 2000). In this tradition, prior studies indicate multiple pressures drawing families away from pursuing a diagnosis, including the stigma of a diagnosis, immutability of a “life-long diagnosis,” and the desire to defend the status of their child as “typically developing” or holding a language delay that will resolve (Ryan and Salisbury 2012; Midence and O’Neill 1999). In contrast, research also suggests pressures and processes that advance parents towards an ASD diagnoses, including diagnostic resolution, parent absolution, and provision of additional resources and professional advice (Russell and Norwich 2012). Russell and Norwich (2012) conceptualize the tensions that emerge from these pressures as a balancing act, in which a tipping point is reached over time during the pre-diagnostic experience leading families to pursue diagnoses (or not) (Russell and Norwich 2012).

Prior research suggests that parents detect developmental concerns for children with ASD as early as 12 months and ASD diagnoses can reliably occur at 2 years of age (Kleinman et al. 2008; Lord et al. 2006; Kozlowski et al. 2011; Bolton et al. 2012). However, the average age of diagnosis of ASD for children in the United States is between 3.5 and 5 years old (Department of Developmental Disabilities 2015). Multiple barriers lead to delays in diagnosis, including extended wait times for patients to see providers (Bisgaier et al. 2011) and shortages of qualified providers (Basco and Rimsza 2013). While this line of research highlights the salience of multiple pressures acting upon families, it provides less insights into how the extended periods of time required for ASD screening and diagnostic evaluation influence the ways within which families make sense of potential differences from a “typically developing child.”

Rather than engaging a “stress-reaction” or “loss” paradigm, which highlights the impact of the diagnosis on family members, the present study engages a constructionist perspective (Best 2017). Constructionist perspectives draw attention to how we co-construct our subjective understanding of the world and reality based on dynamic interactions with other individuals and groups (Conrad and Barker 2010). A constructionist approach does not make claims about the physical reality of disability, but rather examines the “web of significance” created by the individuals in dialogue about a given problem (Griffith et al. 1990; Geertz 1973). Constructionist analyses specifically examine how a problem is constructed and the ways within which available frameworks both provide and constrain how individuals think and talk about the phenomenon of interest (Conrad and Barker 2010).

For the present study, our constructionist approach draws attention to the screening process for ASD as a “framing event” that holds the potential to transform perceptions of specific behaviors into potential ASD symptoms with an associated set of understandings regarding etiology and prognosis. In medical and psychological understandings, ASD is defined as a pervasive developmental disability that is characterized by persistent and pervasive deficits in social communication and social interaction and restricted, repetitive patterns of behavior, interests, or activities, that exist along a spectrum (American Psychiatric Association 2013). In prior studies of children with ASD, researchers investigated how parents integrate the internal representation of their self as parent and the child prior to and after diagnosis (Midence and O’Neill 1999). This research considers how new representations may emerge to facilitate congruence with the new reality of having a child with special needs rather than a “typically developing child” (Abbott et al. 2012). Studies refer to the extent of reconciliation between these divergent representations as a “resolution of diagnosis” (Marvin and Pianta 1996). In these studies, resolution is defined as when a parent accepts both the difficult and positive aspects of the diagnosis and its implications. Furthermore, these studies have employed retrospective investigations that focus on the pre-diagnostic experience of those families who have already received an ASD diagnosis. Studies on resolution of diagnosis focus on the parent’s emotional and cognitive appraisal of the diagnosis and related experiences, and the strategies subsequently employed to cope with the new and unexpected circumstance (Milshtein et al. 2010; Abbott et al. 2012). The present study includes parents whose children participate in screening for ASD but only some of whom’s children receive such a diagnosis; the heterogeneity of our sample facilitates holistic understanding of how the process of screening and assessment creates new meanings for parents with varied experiences. This line of work provides new insights into the experiences of those families whose children are lost to follow-up during the screening process; such insights are critical to understanding how administration of an ASD screening protocol may influence sustained engagement or attrition in the diagnostic process.

The U.S. Preventive Services Task Force report highlights the urgency of evidence being made available on experiences associated with universal ASD screening, specifically concluding “Although there is limited evidence about the harms of screening for ASD in children, reported potential harms include misdiagnosis and the anxiety associated with further testing after a positive screening result…”(Siu et al. 2016, p. 693). Moreover, the report highlights the “screening had a high dropout rate between screening steps and between screening and diagnosis, suggesting that the process may be difficult for some families” (Siu et al. 2016, p. 695). By studying the experiences of families that do not proceed to an ASD diagnosis as well as those that do, our study is better positioned to consider the heterogeneity of parental experiences during universal screening. More specifically, we were able to examine how screening influences the meaning parents attach to their respective child’s behaviors when screening does not result in a diagnosis. Investigation of how parents come to understand concerning behavior during extended periods of screening and assessment is critical in building an evidence base responsive to the high rates of attrition documented in ASD screening programs.

Prior studies of parental perception of ASD screening also tend to be cross-sectional in nature, asking families to reflect upon a pre-diagnostic experience in many cases months or even years after diagnosis. Due to recall bias, these studies are limited in their ability to investigate the screening process, itself, and the dynamic and evolving process families engage in over the extended period of time required for screening and assessment of ASD. The present study is longitudinal in nature allowing for us to investigate how families make meaning of their child’s behaviors throughout screening and assessment of ASD; we leverage this study design to examine the trajectories of families in coming to understand their child’s behaviors through a screening and assessment process.

## Methods

This qualitative study was conducted as part of a community-based research project in partnership with three Part C Early Intervention agencies that utilized a Type II effectiveness-implementation hybrid approach to reduce health disparities in access to ASD services in a Northeastern city of the United States (Curran et al. 2012). Qualitative methods were employed to acquire an in-depth understanding of how the process of screening shapes parental perceptions of their child’s behaviors (which includes both behaviors that parents are concerned about as well as behaviors they report that professionals may find concerning) (Schwandt 1994). This study engages a longitudinal study design, interviewing the same parents 1–6 times to learn about whether and how a multi-stage screening process dynamically and incrementally influences parental perceptions of a child’s behaviors. This research was conducted by an independent team of investigators who were not the interventionists but worked in concert with them. All study procedures were approved by the institutional review board at University of Massachusetts Boston and informed consent was documented in writing.

### Screening Protocol

All parents described in the present paper were referred to and enrolled in Early Intervention (EI) services through Part C of the Individuals with Disabilities Education Act based on developmental/behavioral concerns of the child, qualifying conditions (e.g., Preterm delivery, Trisomy 21), or environmental risk. After enrollment in EI services, the parent respondents participated in at least the first stage of a two-stage ASD screening protocol that facilitated a university-based, research diagnostic evaluation. All screening was conducted as part of routine clinical care by early intervention specialists in participating agencies. During Stage 1, parents completed two screening instruments: (1) the Brief Infant Toddler Social Emotional Assessment (BITSEA) and (2) the Parent’s Observations of Social Interactions (POSI). The BITSEA is a global screener of social emotional development (Briggs-Gowan et al. 2002) that includes two ASD-risk indices, which sum values from responses for 10 problem behaviors relevant to ASD (ASD Problem Scale) and for 9 ASD-relevant competencies (ASD Competence Scale); the resulting scores display high accuracy in detecting ASD diagnoses (Kiss et al. 2017). The POSI is a second ASD-specific screener that has demonstrated strong sensitivity and adequate specificity in two previous studies (Salisbury et al. 2018; Smith et al. 2013). This first stage screening protocol was specifically designed to include two screening tools, thus effectively minimizing the occurrence of false negatives (Eisenhower et al. 2020). Stage 1 participants would advance to Stage 2 if the parent provided consent to continue and either the (1) BITSEA or POSI indicated concern, (2) the parent indicated ongoing concern, and/or (3) the provider indicated ongoing concern. During Stage 2, a trained EI provider administered a 20 min, play-based observational assessment, called the Screening Tool for ASD for Toddlers and Young children (STAT) (Stone et al. 2000, 2008). Stage 2 participants would advance to a diagnostic assessment if the parent provided consent and either the (1) STAT indicated concern, (2) the parent indicated ongoing concern, or (3) the provider indicated ongoing concern. Participants could therefore from the Stage 1 to Stage 2 screening and then through to the diagnostic assessment based on a number of combinations between indicated concern from the screening tool, provider concern, and/or parental concerns. Stage 1 and Stage 2 screenings were administered by EI providers as part of routine clinical practice, whereas diagnostic assessments were conducted by clinical research staff at a university clinic. The diagnostic assessments included the Autism Diagnostic Observation Schedule, Second Edition (ADOS-2), (Lord et al. 2000) the Mullen Scales of Early Learning, (Mullen 1995) parent interview form of the Vineland Adaptive Behavior Scales, Third Edition, (Sparrow et al. 2016) and a semi-structured developmental and medical history interview. Final DSM-5 ASD diagnoses were assigned based on the licensed clinician’s evaluation after observing the visit and reviewing all of the diagnostic evaluation results.

This intervention model was developed to capitalize on the family-centered, home-based nature of Early Intervention services (Eisenhower et al. 2020). The administration of these screening tools is intended to open opportunities for parent-provider conversations about the child’s behaviors and to improve early detection and treatment of ASD for this high risk and historically underserved population. To capitalize on the family-centered approach of EI, the Stage 1 screeners are typically administered by the service coordinator for a child, who also provides the child’s weekly services, enabling ongoing conversations to take place along the way. The Stage 2 screeners and diagnostic assessment are typically observed by the same EI service coordinator facilitating continuity and ongoing conversation as families move through the two-stage screening and diagnostic assessment process.

### Recruitment

To participate in the present qualitative study, parent respondents who had participated in any part of the screening process described above, were recruited through the three EI agencies and engaged in the multi-stage screening intervention. In this article, “parent” is used to refer to the psychological parent of the child such that our use of parent includes biological and non-biological caregivers engaged in the parenting role. The three sites were located in an area of a Northeastern city, where 98% of children come from racial/ethnic minority populations and 47% are English language learners. Two researchers presented the opportunity for parents at their respective EI agencies to complete a series of longitudinal interviews concurrent with the multi-stage screening process. After the introductory meeting, EI staff were provided informational packets to distribute to eligible families; the packet included a “consent to contact” form that would be completed by interested parents. Eligible families for this study had to proceed at least to the Stage 2 screening due to a positive screen, indication of parental concern, or indication of provider concern and be Spanish- or English-speaking. If completed, a member of the qualitative research team contacted the parent and coordinated a time to complete the interview at a quiet location most convenient for respondents. Interviews occurred at the family’s home and at the EI agencies. Parent respondents completed informed consent prior to study participation and received a $40 gift card and reimbursement for associated travel at the end of each interview. To support longitudinal data collection, the research team maintained ongoing engagement with the parents after establishing initial contact.

### Participants

To build new understanding of how families experience a multi-stage screening protocol, we conducted 63 longitudinal semi-structured interviews with 22 parents among 19 families engaged in the screening protocol outlined above. Interviews were conducted at each stage of the screening process in which the family participated, and a final exit interview was conducted if they did not proceed with Stage 2 screening or the diagnostic assessment. Longitudinal interviews ranged from 1 to 6 interviews per family over as long as a year and a half. Families were eligible to participate in the screening protocol if their children (a) were enrolled in EI, (b) had no previous diagnosis of ASD, (c) had no medical condition that would limit the ability to diagnose ASD, (d) were between the ages of 14 and 33 months, and (e) a parent or guardian understood Spanish or English sufficiently to complete the study screeners. All parent respondents participated in at least the first stage of a two-stage ASD screening protocol that was administered within the Part-C EI system and which facilitated a university-based, research diagnostic evaluation. Accordingly, all families received IDEA Part-C family-centered services, irrespective of participation in this study, to address problems in any of five domains: speech and language, social emotional skills, motor skills, adaptive, and cognitive development.

**Fig. 1:**
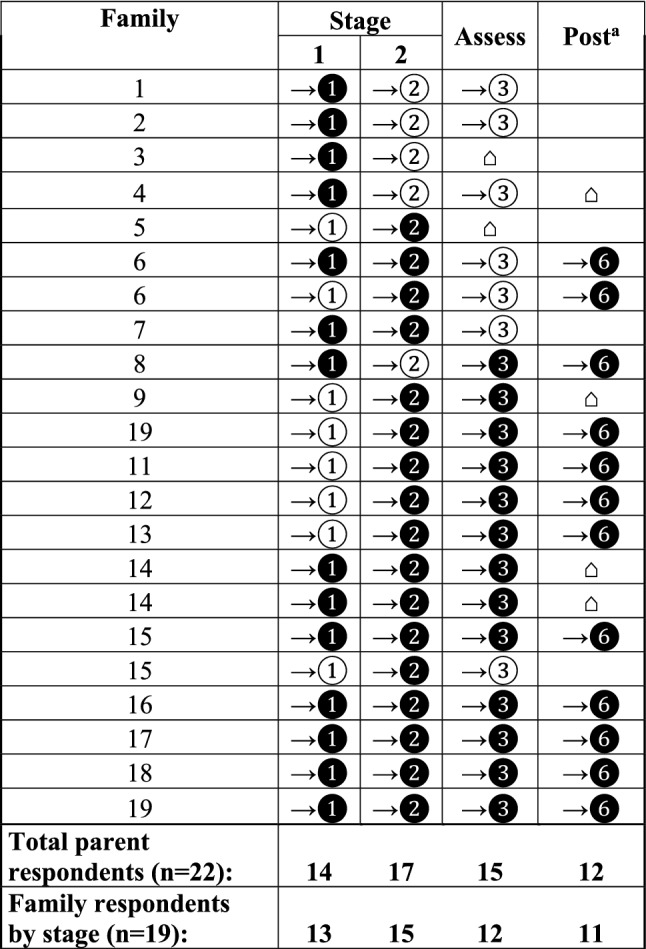
Parental participation across screening and diagnostic assessment stages and 6-months’ post assessment (post). Denotation of “ → ①”in the Stage 1 column indicates families completed the Brief Infant Toddler Social Emotional Assessment BITSEA) and Parent’s Observation of Social Interaction (POSI) while “ → ❶” indicates completion of both the BITSEA/POSI and the interview before advancing to Stage 2. The same convention is used after completion of the Screening Tool for Autism in Toddlers and Young Children (STAT) at the end of Stage 2 when indicating “ → ❷” and “ → ②" and after the diagnostic assessment (Assess), indicated as “ → ❸,” and 6 months after the diagnostic assessment (Post), indicated as " → ❻." Denotation of “⌂” indicates when a family completed an exit interview and exited the screening process. ^a^Post refers to 6 months after the diagnostic assessment for Autism Spectrum Disorders was conducted

Figure 1 indicates how far families proceeded in the multi-stage screening protocol and whether there was an interview conducted at the end of each stage. As noted in the figure, a solid circle indicates that the child of the parent respondent completed that stage of screening and the parent participated in the interview. A clear circle indicates that the child of the parent respondent completed the screening or assessment stage, but the parent did not complete the associated interview. Of the 19 families, 14 families completed the Stage 1 interview. Five families did not complete the interview after Stage 1 because the process of recruitment into this qualitative arm of the study took longer than the time required to schedule and complete the Stage 2 screening. In addition, 2 families (1 and 4) completed the screening process twice and completed 3 additional interviews that are not represented in the figure. Therefore, the total number of interviews completed and coded was 65. Notably, 2 families continued in the screening process after the first interview but 1 family declined to participate further in the study and the other was lost to follow-up.

A total of 19 families were included in this sample, composed of 22 parents. Consistent with the goals of the parent study to improve access for racial/ethnic minority groups, the majority of the parents (69%, n = 15) reported being a racial/ethnic minority and approximately three quarters reported high school degree as the highest attained (49%, n = 9). The majority of children were also reported to be racial/ethnic minorities (84%, n = 16) and to hold public insurance (79%, n = 15). Of the 19 children engaged in the multistage screening protocol, 13 (68%) received an ASD diagnosis. Over the course of the longitudinal interview, families were eligible to participate if their child was between the ages of 14 and 39 months. Additional sociodemographic characteristics of the parents, and sociodemographic and clinical characteristics of the children are provided in Table 1.

**Table 1:** Parent participant and child characteristics

Variable	n (%)
*Parent participant sociodemographic characteristics (n = 22)*	
Parent gender	
F	19 (86)
M	3 (13)
Parent race/ethnicity	
Non-Hispanic white	7 (31)
Non-Hispanic black	4 (18)
Hispanic black	3 (14)
Hispanic Latino (not Hispanic black)	4 (18)
Asian	1 (5)
Multiracial	3 (14)
Parent education level	
Some high school	2 (9)
High school	7 (32)
Some college	7 (32)
Associate or Bachelor’s degree	5 (23)
Master’s degree	1 (4)
Parent reported annual income	
$0–$15,000	10 (46)
$15,001 to $25,000	1 (4)
$25,001 to $35,000	2 (9)
$45,001 to $55,000	3 (14)
$100,001 to $125,000	5 (23)
Not reported	1 (4)
*Child sociodemographic and clincial characteristics (n = 19)*	
Child gender	
F	3 (16)
M	16 (84)
Child race/ethnicity	
Non-Hispanic white	3 (16)
Non-Hispanic black	4 (21)
Hispanic black	3 (15)
Hispanic Latino (not Hispanic black)	4 (21)
Asian	1 (5)
Multiracial	4 (21)
Child insurance	
Private	4 (21)
Public	15 (79)
Parent-reported reason for child’s receipt of early intervention	
Speech & language	12 (63)
Speech & development	1 (5)
Speech & physical therapy	2 (11)
Speech & behavior	2 (11)
Behavior	1 (5)
Development	1 (5)
Child ASD diagnosis status	
Received ASD diagnosis	13 (68)
No diagnosis of ASD received	6 (32)

Parent sample is 22 and child sample is 19, as three children had both parents participate in the parent interview

## Methods

Brief surveys and an in-person semi-structured qualitative interview protocols were developed by trained qualitative researchers for parents; interview guide domains and measures were informed by those published in the peer-reviewed literature and an inter-disciplinary research team. Parent interviews included both paper-and-pencil surveys of participants’ demographics and in-person semi-structured interviews. Each interview consisted of approximately forty categorical and open-ended questions. First, parents were asked to complete a survey providing (1) sociodemographic characteristics, (Guinchat et al. 2012; Zuckerman et al. 2014a, b) and then were engaged in a semi-structured interview with questions pertaining to (2) perceptions of developmental and ASD concerns, (Guinchat et al. 2012; Zuckerman et al. 2014a, b) (3) perceptions of screening tools and results, (Zuckerman et al. 2014a, b; Calzada et al. 2012), and (4) the process of engagement during the multi-stage screening protocol. Reported in this paper are parental descriptions of the multistage screening process and their perspectives and concerns regarding developmental delay and ASD over the course of the screening protocol and subsequent diagnostic assessment. Our research team previously assessed the comparative utility of different pathways that resulted when a toddler was advanced due to: (1) a positive screening result and parent/provider concern, (2) parent and/or provider concern alone, and (3) a positive screening alone (citation withheld to preserve anonymity). Lasting approximately 45–60 min, each interview was audiotaped and transcribed verbatim. For parents, our ongoing process of data collection and analyses identified thematic saturation on core domains on the parental interpretive processes described below (i.e., no new emergent themes) after approximately 15 parent interviews indicating a sample of adequate size for qualitative analyses presented (Ness 2015).

### Analysis

Qualitative data from semi-structured interviews were analyzed using a modified grounded theory approach known as “Coding Consensus, Co-occurrence, and Comparison,” in which analyses are derived from the data and then illustrated by characteristic examples (Willms et al. 1990). Transcripts were analyzed at the family-level. Therefore, transcripts of three families over time were independently coded by an interdisciplinary team of investigators, including a health services researcher, medical sociologist, and clinical psychologists. Investigators coded excerpts of the transcripts, ranging from a phrase to several paragraphs, based on a priori or emergent themes. Disagreements were discussed and resolved, strengthening the definition of the respective codes. Based on the drafted codebooks, three investigators separately reviewed two additional transcripts to determine level of agreement in the codes applied. The codebook captured text on whether and how parents perceived concerns associated with developmental delay and ASD for each time period for a specific family; the codebook also identified text expressing whether resolutions were attained to continue in multi-stage screening process. A final set of code definitions were then discussed, resolved, and recorded. All transcripts for both providers and parents were then reviewed, coded, and subsequently compared and reconciled by at least two of the investigators. Throughout this process, we used intensive group discussion as our goal was consensus rather than a quantitative measures of inter-rater agreement (Harry et al. 2005). Coded data were entered into Dedoose Version 7.0.23 (2016), a mixed methods software program, and a series of categories for these data generated with links between the categories. Investigators then charted the progression of concern for each family regarding their child’s development. The investigator then reviewed these data and wrote a synopsis for each family’s set of interviews on trajectories of parental concern over time and resolution(s) reached if any; all synopses were supported with illustrative quotes of the coded data. At least two investigators charted all data and a process of consensus generated a final matrix documenting parental trajectories over time in assessing concern and reaching resolution. The themes identified across these interviews (“sensitization,” “differentiation,” and “explication”) reached the standard of thematic saturation and are reported with illustrative examples, citing to the gender of the parent and whether the quote was provided after completing the Stage 1 screening tools (i.e., “Stage 1”), Stage 2 screening tool (i.e., “Stage 2”), or the diagnostic assessment (i.e., post-diagnostic assessment).

## Results

**Fig. 2:**
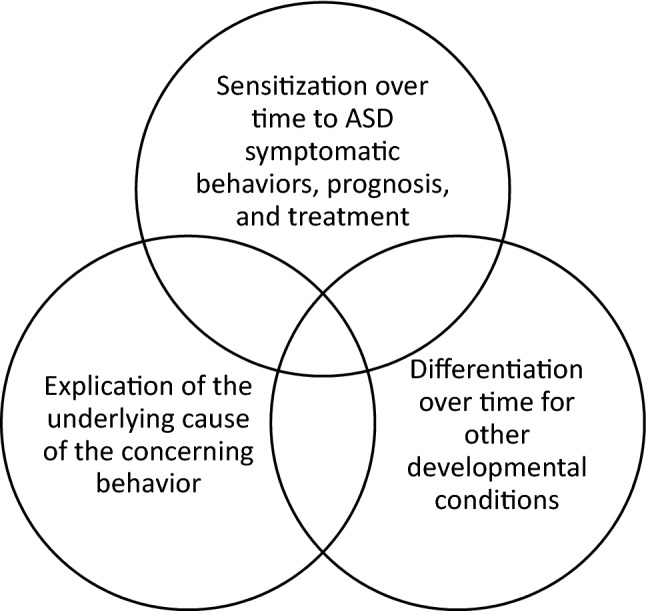
Illustration of the dynamic process of sensitization, differentiation, and explication during ASD screening and diagnostic assessment

Over the course of the screening process, parents expressed engaging in a dynamic process of understanding their child’s behaviors in new ways, moving them towards, away from, or oscillating between an interpretive frame consistent with ASD over time. Our analyses indicate that the dynamic and multiple interactions of the parent with both the screening tools and the EI provider affected the parents’ understandings of their child’s behaviors in multiple ways. In particular, our study specifically finds that parents engaged in three dynamic and bi-directional processes that were influential as parents moved through the screening protocol, specifically (1) sensitization over time to ASD symptomatic behaviors, (2) differentiation over time from other developmental conditions, and (3) explication of the underlying cause of the concerning behavior. Notably, we define “sensitization,” “differentiation,” and “explication” as unique concepts; however, we find these processes may occur simultaneously, as illustrated in Fig. 2.

### Sensitization

So in [the administrator of the screening tool] asking me certain things and me answering certain things it kind of helps me distinguish, oh that’s just two-year-old stuff and that’s something more serious.—Mother, Stage 1.

The screening process for some parents facilitated a process of *sensitization,* defined as shifting families’ understandings of which specific behaviors represent symptomatic presentations consistent with the ASD interpretive frame. Parent frequently explained speech delays or emotional regulation as behaviors of initial concern as illustrated below:

I’m a little concerned about her behaviors. I feel she’s gotten a lot more frustrated, crying all the time…You know, she’s pushing a lot, pulling hair. But again, could be nothing. Could be frustration cuz she can’t talk.—Mother, Stage 1.

Notably, this mother describes behaviors of concern, although associates the concerning behavior as potentially related to speech delays. Sensitization occurred across multiple phases of the screening protocol. After the diagnostic assessment, parents who moved to an ASD interpretive frame described becoming sensitized to a set of behaviors associated with ASD that, as the following parent notes, she “just wasn’t used to seeing…”:

*Interviewer:* …What are some of the other behaviors that she has that are associated with ASD? *Interviewee*: There’s a lot of stimming; there’s different things that she does that they classify as stimming. She does this rocking motion with her stomach and she’ll do that pretty intensely to where she starts to get really clammy and sweaty. That’s usually around the time that she’s ready to go to bed. She’ll kind of rock herself to sleep that way. She walks on her toes, which again I heard is like a muscle pull, so it’s like a stimulant. I don’t know. Her eyes kind of deviate to the side and they’ll stay there for a while, and that is also a muscle pull is what I’m understanding. She does this thing where she kind of focuses on her hand. She’ll stop, like mid-stride of walking somewhere and just put her hand up, tilt her head and just stand there for a little bit, just a focus, and then she’ll put it down and then she’ll keep going. I’m trying to think of anything else. Little things that she does, but those are things that I just wasn’t used to seeing, you know.—Mother, post-diagnosis.

As illustrated above, sensitization occurred for multiple families engaged in the multi-stage screening and assessment process with behaviors not previously identified as “concerning.” Sensitization occurred dynamically over the course of the process reflecting new understandings of the symptomatic presentations associated with ASD.

Both the written- and observation-based screening tools led parents to articulate engaging in a process of sensitization, in which they placed specific behaviors within the interpretive frame for ASD. The excerpt below illustrates how respondents articulated having been sensitized to behaviors potentially associated with an ASD interpretive frame:

*Interviewer:* Okay. So, after you did the questionnaire, did you think about some of his behaviors? *Interviewee*: Oh, yeah. It definitely put certain things where you're like, "He doesn't do that. He doesn't do this." Or, "He does that but in a different way." But it definitely brings certain things to your attention that you don't realize or you knew he did but didn't do the way that they want him to do it or are expecting him to do it…"He doesn't know how to do that." You just – there were just certain things that you just don't pick up on until they break it down for you. It was like those little smaller things that you don't realize he doesn't do. Like, I never picked up on him not pointing because I'm just with him all the time. I'm just kind of have a routine.—Mother, Stage 1.

Notably, parents reported that the tools facilitate sensitization in distinct ways. Parents reported that the written-based screening tool elicited a reflective process of evaluating specific behaviors in relation to symptomatic presentation of ASD, as illustrated below:

[BITSEA/POSI] is useful…because you kind of get to evaluate your own kid. Honestly, we’re grading our kid…I tell you, because honestly you cannot lie to yourself, as a mom… if you put a three when there’s really a two, no. Because you know. The questions you guys ask are very specific, you know?—Mother, Stage 1.

While the written questionnaire engaged the parents’ evaluation of these behaviors directly, parents reported that the observation-based tool provided the opportunity for a third-party observation of a separate evaluative process, which provided distance that facilitated new understandings of their child’s behaviors:

*Interviewer:* Okay. And why do you think [the observations during the STAT] were helpful? *Interviewee:* Because I got to sit back and actually look and see, without me saying nothing, the teacher was doing the play with him. I got to see more of what his other teacher in the past was talking about.—Mother, Stage 2.

Therefore, parents reported that the written and observation-based tools facilitated sensitization in distinct and complementary ways.

Sensitization of behaviors occurred not only through tools, themselves, but also the language and interactions with the EI providers who administered the multi-stage screening protocol. Illustrative of this theme, one parent points to how the language of EI providers sensitized her to the child’s behavior as consistent with an interpretive frame for ASD:

[EI providers] were using the word, stimming. I had to look that up to see what that meant, and once I realized that that was a key word in autism, and then all of these; I started seeing the things that she does listed as some of the things that kids do, and so that’s when I kind of put the two and two together…—Mother, post-diagnostic assessment.

Parents therefore reported a process of sensitization that was co-constructed both by the tools, interactions with the administrators of these tools, and the parent themselves. When providers and parents held discordant interpretations of behaviors as symptomatic of ASD, parents reported that EI providers purposefully engaged the screening process as a strategy to reconcile these discordant interpretations of specific behaviors. Illustrative of this theme, one parent recalls the EI provider recommending the screen to provide additional information:

The EI provider was noticing a few weeks ago he wasn’t really looking at her that well, so she asked me to do the questionnaire at that point. But I do tend to disagree because he makes pretty good eye contact with us and I just don’t know if he just gets shy around people and didn’t want to look at her. So, that kind of prompted her to let’s do a screening on the paper, so we did.—Mother, Stage 1.

Accordingly, the screening process was, in some cases, deliberately employed by the EI providers to facilitate a process of sensitization of specific behaviors within an ASD interpretive frame.

Despite efforts of EI providers to facilitate a process of sensitization, the screening process did not always generate a shared or fully realized co-construction of specific behaviors into an ASD interpretive frame. Illustrated below, one respondent articulates:

And so there are certain things—like he wasn’t doing that specific thing that they were looking for in that activity…because with my son, I know the things that I see that I’m concerned about, but I do not know definitively if the exact things that I think are so concerning are the exact things that they think are concerning.—Mother, Stage 2.

Therefore, sensitization of specific behaviors into a shared interpretive frame for ASD varied across the engaged sample. That is, some parents continued to hold perspectives that differed from those of their EI providers and the screening process more broadly. Lack of concordance frequently emerged due to a lack of sensitization and or a failure of differentiation with other concerning behaviors. As one parent notes, the “natural autism issue” never fully aligned with her perception:

*Interviewee*: Different pretend play and…requesting certain things in which she did good in part of the pretend play and not good in the other. She didn’t do good with requesting things. But again, I think that’s more of a language issue, than natural autism issue. So yeah, so, like I said, it was a pretty, I mean she did do, you know, she was on the closer of normal, the norm. —Mom, Stage 2.

We next turn to how differentiation operated in moving families towards, away from, or oscillating between an ASD interpretive frame.

### Differentiation

I just needed to know what it was about and when I realized it was all about communication and a social thing and not so much neurologically with what she’s going on with Sturge-Weber [syndrome]. It was completely different. I just realized that we kind of have to attack two things now. Like, different ways.—Mother, post-diagnosis.

The screening process for some parents facilitated a process of *differentiation*, defined as shifting families’ understandings of specific behaviors from the interpretive frame of one diagnostic condition to a new and distinct framework, in this case an ASD-specific frame.

Unlike sensitization, the process of differentiation drew upon information about other conditions and in some cases, the services required to treat them. Illustrative of this theme, one parent initially expressed concern regarding “speech delay” and identified the need for treatment to see the problem resolved.

Also, we feel like one thing that did affect him was that we went to audiology appointment probably like a month or two ago to find out he has fluid in both his ears…*.*So he has an appointment coming up June 12th to get those drained, and maybe it'll help him understand or hear more, 'cause when he says words, it's like they sound a little muffled, and then like he puts words together, so he's not hearing the two words that he's supposed to be hearing and stuff… It only certain ways like he actually understands or hear. So I feel like, once that's done, maybe he'll have more understanding and can hear clearer what we're saying and say it back. —Mother, Stage 2.

Therefore, families perceived the need for engagement outside of the ASD-specific screening process to assist in a process of differentiation.

Respondents articulated that the screening process facilitated differentiation between an ASD-specific frame and behaviors consistent with interpretive frames employed for speech, hearing, or other developmental delays (such as Sturge-Weber syndrome, a disorder characterized by a facial birthmark, neurological disorders, and eye disorders such as glaucoma). Illustrative of this theme, one parent speaks to how differentiations were drawn through the screening process between behaviors associated with language delay and ASD and re-engaging in the process when concerns for speech delay persisted and other concerns were raised in addition to the “speech and stuff”:

They explained [ASD] by, like, her speech and stuff that, you know, that [speech and stuff] could be a symptom of it. They seem to see other symptoms at the [diagnostic] evaluation that I have never noticed before….the way she looked at, like, you know, like toys. They said she holds it up to her face and kind of looked to the side, which I had never noticed. You know, her not paying attention when you call her name, which I guess she did at times. —Mother, 6-month post diagnostic assessment.

As illustrated above, the process of differentiation did not always occur in isolation but frequently occurred in combination with other interpretive processes. In the quote above, the respondent associates “speech and stuff” with symptomatic presentation of ASD while also indicating sensitization to other social-communicative symptomatic presentations consistent with ASD.

In contrast, parents in our sample did not differentiate ASD from other conditions for two reasons. First, respondents would, in some cases, not”see” the specific behaviors in their child, such as the presence of social-communicative skills, that were consistentwith the ASD interpretive frame. Illustrative of this theme, one respondent articulated:

I think she, you know, agree, because of the speech, which she is delayed….I mean her strengths, with that being said, are which you don’t usually see in autism from what I hear is eye contact and being social. Those are two of her strengths…—Mother, Stage 1-second round.

Second, respondents also rejected the ASD interpretive frame for specific behaviors when they identified improvements in the specific behavior associated with the ASD interpretive frame. For example, another respondent articulated moving away from the interpretive frame of ASD given improvements observed in social-communicative skills:

*Interviewer:* And do you think—you spoke about this a little bit, but do you think it's a good fit for him, the diagnosis? *Interviewee:* Oh gosh. I guess that's the reason why I want further testing 'cause I feel like it's not…It doesn't fit. Socially, in communicating now it's like—it just clicked. I feel like when he doesn't have the word he gestures, and he's using signs to tell me what he needs. And he'll ask for help and more. He modifies his own sign, and he's even coming up with a couple of new ones, and I'm like, "What does that mean?" So, I feel like he turned the corner….Further down the line I feel like he's gotten better. So, my biggest – I'll say worry – is that, are we giving him the time to develop and grow out of these stages that he's in? Or is he being diagnosed too early?—Mother, post-diagnostic assessment.

The parent above, illustrative of perspectives presented by other respondents, denoted shifting back to understanding the behavior as something capable of being “grown out of,” a characteristic perceived as inconsistent with the ASD-interpretive frame. Accordingly, the behaviors associated with ASD may be understood by families to be immutable leaving families to reframe the behavior in a way consistent with other conditions amenable to improvement (such as speech delay).

### Explication

With him it [ASD] is more towards his speech and him not being able to communicate what he wants to us. At first I’m thinking it’s his age. I can’t understand a word he says because he’s two. He’s not supposed to be talking in full sentences, but also now it’s more he’s at the age where he should be able to say I want this or I want that and he’s not so that’s where we’re kind of seeing it, where I’m seeing it. His dad is not quite there yet.—Mother, post-diagnostic assessment.

Explication is part of adopting an interpretive frame in which ASD is perceived as responsible for the etiology or the underlying cause of certain behaviors. Thus, the ASD interpretive frame is used to explain these behaviors. Parents initially explain the cause of specific behaviors in various ways. For example, parents attributed to behaviors that were later associated with an ASD interpretive frame to the child’s developmental stage, gender, and culture. In other cases, parents attribute the reason for specific behaviors with how and when the screening process itself was administered. For example, parent respondents attributed the presence of specific behaviors to the child’s age, constructing a narrative that a specific behavior would no longer be present as the child developed further.

I just feel like they didn't consider his age—current age and stage development—and that giving him the time to sort of kinda say, "We haven't seen a change…" So I just feel like everything he's done is sort of kind of was a stage where he was stuck on, and once he figured out how to proceed, in other words, he outgrew that current concern. It just, it just kinda felt like he needed some time.—Mother, 6-months post diagnostic assessment.

Not like, I don’t want to sound like that, but it could be just me, too, but I think that’s too young to say that he has this [referring to Autism] or something like that. He’s a kid. It takes a little bit longer for him to develop or something like that.—Mother, Stage 2.

As time passed, some families expressed that the behavior had not changed in ways anticipated, which motivated adoption of an ASD interpretive frame over time:

I didn't think at first that he had any type of disorder because he's two years old, and even before he was two, I pretty much thought that he was doing what babies do…But then as he started getting a little bit older and older, like two and a half, I'm like, "Wait a minute. He's not giving me eye contact. He's really not giving anybody eye contact." So I kinda noticed something is not right.—Mother, post-diagnostic assessment.

Specific behaviors were also associated with the child’s gender. Parents report that additional information about the behaviors shifted the interpretation of specific behaviors as typical for boys to an ASD-specific interpretive frame, such as “hand flapping” and “no eye contact.”

*Interviewee:* Because I think, once I was informed on autism and little like ticks they have or things they do—and not everyone's the same …but I think I paid more attention, because I think a lot of the things I thought—I brushed it off as "He's two and he's a boy and he's hyper." And once I got a little more information, it made me observe him more and I got a little concerned with repetitive things and the hand flapping and no eye contact sometimes.—Mother, post-diagnostic assessment.

Notably, perceived differences in presentation of ASD among genders generated additional questions on understanding behaviors consistent with an ASD-interpretive frame. As one parent articulates:

I mean, I think Child's case is very—it's—besides her confusing because we had been working on it for a long time. And she really doesn't have any true signs…But again, they said that girls can show different than boys.—Mother, post-diagnostic assessment.

Finally, members of our diverse sample also indicated attribution of specific behaviors to the culture and community to which the family belonged. One participant describes the attribution of specific behaviors to their culture:

Like I said, I have friends with kids that walk like that and they're a little different than me. I knew that wasn't right and my friends are kind of like, oh they'll get over it. It's a Spanish thing.—Mother, Stage 1.

Characteristics of the screening process itself, was also used to explain why specific behaviors did not align with the ASD interpretive frame. Parents reported understanding specific behaviors in relation to the context of the screening provided (e.g., time of day/post-nap).

*Interviewer:* Yeah. Okay. So he did the play-based screening. And how did he do on that? *Interviewee:* They feel like he scored very well. And I feel like when they did come, he had just woke up from a nap. So after that he was just like, "I really don't wanna do that. I wanna eat. I wanna watch TV. I don't wanna do anything." *Interviewer:* Okay. *Interviewee:* He did some of the stuff here and there, but when it came to certain toys—like he loves cars. When they brought out the car, he wasn't trying to roll it back. He was like, "No, I wanna hold onto this one the whole time." So it wasn't like he was following the steps that they wanted him to follow, so they kinda like failed him for that 'cause he wasn't playing along with them and stuff. *Interviewer:* Of course. Of course. So, at this point, what are some of CHILD's strengths? What is he doing well? *Interviewee:* I feel like – hmm. I feel like he does well with – I feel like he does more well when he's in school. *Interviewer:* Okay.—Mother, Stage 2.

Parents also indicated that the screening process failed to take into unique aspects of the child’s development and behaviors, including temperament in working with “strangers,” and specific ways of communicating. In each case, the parent offers a different explanation challenging the application of the ASD-interpretive frame for the behavior. Below, one parent articulates a different interpretation of behaviors moving her further from understanding the behaviors consistent with the ASD interpretive frame:

Then this is where I feel that—I feel like they're wrong because he did certain things that for them it was like he's doing a repetitive behavior. And it's like, "No, no. If he's doing this. It's not repetitive. It's a vehicle. It's a truck. It's a train. It's a car." He generalizes. He uses this gesture to generalize. So, he's going to use it more than once to mean a specific thing. *Interviewer:* The gesture of waving his hands. *Interviewee:* He does the wheel. This means wheel to him, so if he sees a train he'll see wheel. And if he sees the car underneath the couch he'll say wheel. If he sees something that looks like a wheel, he'll say wheel. But, it's not a repetitive. There's a purpose why he's doing it. So, for them, it was like, "Oh, it's a repetitive behavior." It's like, "No, it's not. It's just his way of communicating that he sees a truck and a plane. 'Cause he's signing.—Mother, post-diagnostic assessment.

In this quote, the parent explains how she expressed that the interpretations of specific behaviors as repetitive did not align with her own interpretation of that same behavior. Therefore, efforts to explain the reasons for a child’s behaviors were bi-directional in some cases moving the families toward an ASD diagnosis and yet in others moving them further away.

## Discussion

Our findings detail how parents report making meaning of specific behaviors that could indicate ASD symptoms and concerns presented during an ASD screening process. Prior research suggests parents of children with ASD are challenged to reconcile or reach resolve between the internal representations of a “typically developing child,” or a child with other special needs such as a language delay, and those for a child with ASD (Abbott et al. 2012). However, limited research to date characterizes how the screening process may facilitate or impede the ability for a family to adopt a framework for understanding behaviors in a manner that is consistent with the ASD interpretive frame. Our study finds the screening process consistently engaged families in reconsidering their understanding of their children’s behaviors within the ASD interpretive frame. New understandings arrived to the families regarding the symptomatic presentation, etiology, and prognosis for ASD. As noted in the findings reviewed above, families varied in the extent to which this new information moved them towards an ASD interpretive framework for their child.

Our research identified three dynamic and bi-directional processes that were influential as parents moved through the screening protocol, specifically (1) sensitization to ASD symptomatic behaviors, (2) differentiation of ASD symptomatic behaviors from other developmental conditions, and (3) the use of the ASD diagnostic framework to explain certain patterns of behaviors (explication). First, families expressed that both screening tools and clinical interactions sensitized them to behaviors within an ASD interpretive frame that they had not previously thought warranted concern. Second, families frequently spoke to the clinical complexity exhibited by their child (i.e., multiple co-morbid conditions) and how the screening facilitated differentiation between other developmental conditions and ASD. Third, families held concerns for specific behaviors and, in some cases, shifted attribution or explanations as to the cause(s) for these behaviors from factors related to the child/family or the screening process to ASD.

Our research brings new evidence to these concerns, suggesting efforts to promote sensitization, differentiation, and explication may assist families in conceptualizing their child’s behaviors within an ASD interpretive framework and facilitate the diagnostic process. Notably, dynamic interactions with both written and observation-based screening tools facilitated sensitization to specific behaviors within the ASD interpretive frame in distinct ways. The written questionnaire engaged the parents’ evaluation of these behaviors directly and facilitated reflexivity and conversation (with the administrator) on whether congruent understandings of the child’s behaviors existed. In contrast, parents reported the benefit of being a third-party observer during the observation-based screening tool; this screening approach allowed for real-time observations of discrete activities that facilitated, for some parents, additional distance and focus required to understand new behaviors as ASD symptomatic. These findings would suggest that the use of multiple types of screening tools (written and observation-based) may be more likely to influence parents in moving towards an ASD interpretive frame by generating complimentary processes for increased sensitivity to symptomatic behaviors consistent with an ASD interpretive framework and potentially improve retention of families through to diagnostic evaluation and treatment. Future studies to test the hypothesis that multi-stage screening protocols do improve sensitization and ultimately retention to ASD-specific services are warranted.

The U.S. Preventive Services Task Force highlighted the urgency of evidence being made available on experiences associated with universal ASD screening given the limited evidence on harms associated with screening and “high dropout rate between screening steps” (Siu et al. 2016, p. 695). Prior literature largely focuses on the “diagnostic journeys” of families whose children had already received an ASD diagnosis. The multi-stage screening protocol implemented in this study not only included those who had never received an ASD diagnosis, but also included provisions for toddlers to be re-screened after the initial screening (i.e., 6 months or dependent on indicated concerns). For two families in our sample, this led their child to receive an ASD diagnostic evaluation twice. One of these families did not receive an ASD diagnosis after the first evaluation but met clinical criteria for ASD the second time. Notably, this generated a less linear path in moving the parent toward the ASD interpretive framework. The parent reported engaging in a dynamic process of sensitization and de-sensitization in their first engagement with the screening protocol although ultimately arrived at an ASD interpretive frame. Despite diverse trajectories and experiences of parents in our sample, our analyses identified a common set of processes when families moved towards an ASD interpretive frame, specifically sensitization, differentiation, and explication.

During the process of differentiation, parents notably indicated a need for concomitant screening of other concerning behaviors to distinguish between ASD and other potential diagnoses. This finding suggests that screening for ASD benefits from coordination and attention to other developmental conditions (e.g., speech delay) that may be understood by parents to cause the behaviors that clinicians associate with ASD. For example, explaining that many children with ASD have language delays and then highlighting specific aspects of language that are not only delayed but that are atypical and consistent with the pattern of language development observed among children with ASD (e.g., unusual intonation, repeating sound combinations or phrases from books or videos) as well as highlighting repetitive behaviors that are not commonly observed among children with language delays may aid in sensitization, differentiation, and explication. Indeed, orienting parents to specific symptomatic behaviors in real time and wondering with them about how they understand the child’s behaviors can help create alignment between parents and providers.

Another implication of our findings is that moving families through these dynamic processes towards an ASD interpretive frame may require additional time for providers to work with families. Our longitudinal analyses demonstrate that ongoing dialogue occurred across the multiple stages of the screening protocol, which in turn facilitated a process of sensitization, differentiation, and explication incrementally over time. This finding is particularly noteworthy given that measured diagnostic processes are more likely than “speedy diagnoses” to be associated with diagnostic resolution among parents (Reed et al. 2019; Yirmiya et al. 2015).

Notably, our findings arrive from a community-based multi-stage screening protocol in a socially-disadvantaged community and specifically sought to reduce disparities in screening participation and outcomes (Sheldrick et al. 2019; Mackie et al. 2020). To examine whether disparities in screening participation and outcomes persisted in the parent study, our research team investigated the predictors of screening participation and outcomes at each stage of the process; demographic differences (race, language, public insurance) were observed only at first-stage screening and reflected higher participation for children of color and higher positive screens for publicly-insured children (Eisenhower et al. 2020). Thus, the intervention demonstrated redress to the well-documented racial/ethnic disparities in screening participation and retention. Future qualitative research, with an adequate sampling framework by the sociodemographic characteristics of interest (e.g., race, language), might also examine whether and how incremental screening processes may facilitate these dynamic interpretive processes in ways that generate engagement and retention among specific socially disadvantaged groups.

As screening can facilitate diagnostic differentiation for families, the value of a screening protocol may not be confined to the identification of behaviors previously unrecognized as symptoms (i.e., the process of sensitization), but may also contribute to the process of differentiating between ASD and other developmental conditions (i.e., differentiation). Screening may thus have utility even for cases where a child presents with clinically-significant symptoms that already evoke concern, but the parent and/or provider do not perceive those symptoms through an ASD interpretive frame. This finding holds significant policy implications. Specifically, the U.S. Preventive Task Force prioritizes evidence that screening is effective among asymptomatic children (Siu et al. 2016), presumably under the assumption that the value of universal screening is restricted to the detection of cases that were previously unknown and for which no concern had been expressed. However, our data suggest that screening can have utility for children with symptoms by familiarizing families with an ASD interpretive frame. Therefore, a full evaluation of the benefits of a universal screening program would benefit from evidence as to whether and the extent to which screening facilitated differential diagnosis and/or reduced the time to diagnosis.

This research study also provides a methodological innovation, by conducting prospective longitudinal interviews to examine parental trajectories during ASD screening and diagnostic evaluation (when applicable). While qualitative studies of the ASD “diagnostic” journey routinely indicate dynamic and non-static perceptions of ASD, (Ryan and Salisbury 2012; Midence and O’Neill 1999; Abbott et al. 2012; Elwyn et al. 2010; Braddock et al. 1997) few studies to date engage in prospective longitudinal interviews to examine trajectories of parental perception of child’s behaviors over time. By doing so, our findings move away from a point-in-time interpretive framework for ASD to instead characterize three interpretive processes in how parents moved to or away from understanding their child’s behaviors within an ASD interpretive framework. Moreover, the present study also suggests that the extent of sensitization, differentiation, and explication during the screening program may influence whether a parent moves towards or away from an ASD interpretive frame (Milshtein et al. 2010; Abbott et al. 2012). Therefore, efforts to facilitate diagnostic resolution for families may require collaboration in engaging screening programs that promote these interpretive processes.

Our qualitative research method is limited in the generalizability of findings outside of the sample engaged. Similar to prior studies of parental experience of ASD, our study disproportionately represents the experiences of mothers. However, interviews were conducted until data saturation was reached, in that no new data repeated what was expressed in previously collected data. Therefore, the qualitative standard for concluding data collection was achieved for this particular sample. The window of time between the screening tools’ administration and our interview varied across participants; this introduces variation in the extent of recall bias across study participants and interviews. However, we did not conduct any interviews that extended beyond 3 months after the index event (e.g., Phase 1 screening, Phase 2 screening, etc.). Our analyses achieved a standard of thematic saturation and crystallization of findings across respondents who described processes of “sensitization,” “differentiation,” and “explication” in moving towards or away from the ASD diagnostic frame. Notably, other interpretations are available for the analyzed data; for example, one might consider the data in relation to health literacy or acquisition of ASD-specific knowledge, attitudes or skills. However, our analytic approach found dynamic processes of sensitization, differentiation, and explication to capture most accurately our empirical findings on families’ trajectories in moving towards or away from an ASD interpretive frame. Given that our sample only included the families’ perspective, our data are not well situated to comment on whether the front-line administrators engaged in a process of sensitization, differentiation, and explication. However, future study is warranted on whether these same mechanisms (sensitization, differentiation, and explication) operate as clinicians engage in screening protocols and with the family’s perspectives and expressed concerns. Finally, our paper does not explicitly allow for analysis of how these processes varied dependent upon whether diagnostic misclassifications had occurred or if the toddler truly met clinical criteria for ASD. As articulated by Singer and Willett, “each observed score is just a fallible operationalization of an underlying true score” (Singer and Willett, 2003, p. 28). Our qualitative study was not designed to detect whether screening results reflect the underlying true diagnosis of ASD. Accordingly, we limit our analyses to whether parental perspectives aligned with an ASD interpretive frame rather than explicitly engaging in whether these perspectives were aligned with an underlying true score for ASD.

Our study employed prospective longitudinal interviews facilitating the ability to investigate the interpretive process by which parents make new meanings of their child’s behaviors over time. We specifically find that families engaged in (1) sensitization to ASD symptomatic behaviors, (2) differentiation from other developmental conditions, and (3) the use of the ASD diagnosis to explain certain patterns of behaviors (i.e., explication). By identifying these interpretive processes, distinct opportunities become available for screening administrators and developers to help families understand behaviors within ASD interpretive frames and to facilitate adaptive protocol designs. Strategies to facilitate these interpretive processes might include efforts to: diversify the screening tools employed (written- and observation-based), use multi-stage screening methods, enhance shared decision-making through conversations about current meanings attributed to child behaviors by parents and clinicians during screening administration, and collaborate with other developmental and mental health screening/assessments to facilitate differentiation.
